# Digoxin in management of heart failure in children: Should it be continued or relegated to the history books?

**DOI:** 10.4103/0974-2069.58317

**Published:** 2009

**Authors:** Shreepal Jain, Balu Vaidyanathan

**Affiliations:** Department of Pediatric Cardiology, Amrita Institute of Medical Sciences and Research Centre, AIMS Ponekkara P.O, Kochi, Kerala, India

Cardiac glycosides have played a prominent role in the therapy of congestive heart failure since William Withering codified their use in 1785.[1] In view of its narrow therapeutic index, limited published data on efficacy in children, and the widespread availability of newer drugs like angiotensin-converting enzyme (ACE) inhibitors and beta blockers, many clinicians have started questioning the role of digoxin in the present day management of heart failure in children. This review and opinion poll focuses on the role of digoxin in the contemporary management of pediatric heart failure.

## MECHANISM OF ACTION

The combined inotropic-bradycardic action is unique for digoxin compared to all other sympathomimetic inotropes that cause tachycardia. Its principal mechanism of action is by the inhibition of the intrinsic membrane protein Na^+^-K^+^ ATPase (a sodium pump), which in turn promotes calcium influx by a sodium-calcium exchange mechanism.[2] This effect is responsible for the inotropic and electrophysiological effects of the drug. Digoxin also causes parasympathetic activation, which results in sinus-slowing and atrioventricular (AV) nodal inhibition. In patients with heart failure, digoxin reduces plasma renin activity and norepinephrine and serum aldosterone levels, and induces diuresis.[3]

## PHARMACOKINETICS

Digoxin is excreted exponentially by the kidneys, with an elimination half-life of 36 to 48 hours in case of normal renal function.[2] The elimination half life may be as high as 61–170 hours in premature neonates.[4] Starting therapy with maintenance doses results in steady-state plateau concentrations after 4 to 5 half-lives (7–10 days) in subjects with normal renal function.[2] In patients with renal failure, the volume of distribution of digoxin is decreased, necessitating reduction in the loading as well as maintenance doses.[5] Digoxin crosses the placenta, and fetal umbilical cord venous blood levels of the drug are similar to maternal blood levels. About 30% of the drug is excreted by the fecal route and hepatic metabolism.[6]

## CONTRAINDICATIONS

Contraindications to the use of digoxin include hypertrophic obstructive cardiomyopathy, antidromic tachycardia through accessory bypass tracts in Wolff-Parkinson-White syndrome, and high-grade AV block. It needs to be used with caution in patients with renal failure, hypokalemia, myxedema, acute myocarditis, premature infants with impaired renal clearance, and co-administration with drugs inhibiting AV conduction (beta-blockers, amiodarone, verapamil, diltiazem). In these settings, dose reduction of the drug is appropriate.

## DRUG INTERACTIONS

Drugs like quinidine, amiodarone, and propafenone increase serum digoxin levels by reducing its clearance. Diuretics may induce hypokalemia which may precipitate digoxin toxicity when co-administered. ACE inhibitors may precipitate renal failure (especially in patients with left-right shunts), thereby increasing serum digoxin levels.

## DIGITALIS TOXICITY

Digoxin has a narrow therapeutic range and side effects are seen in 10–20% of all cases.

Features of cardiac toxicity include sinoatrial and atroventricular blocks, atrial and nodal ectopic beats, atrial tachycardia with AV block, as well as ventricular arrhythmias including bigeminy, trigeminy, and ventricular tachycardia. Heart blocks are more common in children; ectopy is more often seen in adults. Noncardiac side-effects include gastrointestinal (anorexia, nausea, diarrhea), neurological (lethargy, confusion, vertigo, delirium), ocular (blurred vision, diplopia), and tinnitus.

Heart rate and rhythm should be monitored when initiating digoxin therapy, during rapid digitalization, or when the patient is acutely sick. In case of suspected overdose, a sample for digoxin levels should be taken at least six hours after the dose has been administered. Therapeutic range is 0.8–2 ng/mL; toxicity is usually seen at > 2 ng/mL level. Most cases of mild toxicity respond to temporary withdrawal of the drug. For life-threatening digitalis toxicity, the current first-line management strategy is the administration of digoxin-specific antibody fragments. Ventricular tachycardia may be managed with potassium administration and drugs like Lidocaine or Phenytoin. Sinoatrial arrest and high-grade AV block can be treated effectively with atropine, although pacing may be required.

## PREPARATIONS AND DOSAGE

Digoxin is available as an Elixir (60 mL suspension, 50 mcg/mL) and tablet (0.25 mg tab). Tablets should not be crushed to formulate liquid preparations for children. Injectable digoxin (100 and 250 mcg/mL) is available for intravenous use; intramuscular route is not recommended.

The recommended dosage schedule for children is summarized in Table 1.[7] For rapid digitalization, the total digoxin dose is almost four times the maintenance dose. Half of the total digitalizing dose is given after checking the ECG and electrolyte levels; one fourth is given after six hours, and another fourth after another six hours. Rapid digitalization is usually not indicated when using digoxin for heart failure, except for failure associated with tachyarrhythmias. The maintenance dose is given in twice-daily doses for children under ten years of age and once-daily for children above ten years of age. Digoxin “holiday” is generally not needed in children.

**Table 1:** Dosing for digoxin in infants and children[7]

Age	Total digitalizing dose mcg/kg/24 h		Daily maintenance dose mcg/kg/24 h	
	PO	IV	PO	IV
Premature	20	15	5	3–4
Full term	30	20	8–10	6–8
< 2 yr	40–50	30–40	10–12	7.5–9
2-10 yr	30–40	20–30	8–10	6–8
> 10 yrs /adults	0.75–1.5 mg	0.5–1 mg	0.125–0.5 mg	0.1–0.4 mg

PO - Per oral; IV - Intravenous

## CLINICAL TRIALS OF DIGOXIN

Several trials conducted in the adult population have documented symptomatic improvement, increased exercise capacity and total body O_2_ consumption on exercise, improved hemodynamics and ejection fraction, and reduced incidence of clinical worsening of heart failure.[8‐13] These included the prospective trials, PROVED (The Prospective Randomized study Of Ventricular failure and the Efficacy of Digoxin) and RADIANCE (Randomized Assessment of Digoxin on Inhibitors of the Angiotensin-Converting Enzyme).[12,13] The Digitalis Investigation Group (DIG) trial is the largest clinical trial of digitalis in adult heart failure.[14] In this trial, 6800 patients with LVEF ≤ 0.45 were randomly assigned to be treated with digoxin or placebo. The placebo group received diuretics (82%) and ACE-I (95%) and the digoxin group received digoxin, diuretics (81%), and ACE-I (94%). The main findings of this trial included:

1. No effect on total mortality rates
2. Reduced incidence of death or hospitalization caused by worsening heart failure
3. Slight increase in risk of arrhythmic deaths
4. Benefits were incremental to use of diuretic and ACE-I

Recent evidence from studies in adults suggest that higher serum digoxin concentrations (> 1.2 ng/mL) are associated with higher mortality, higher rates of hospitalization, and greater toxicity.[15]

There are no randomized control trials using digoxin in the management of pediatric heart failure. Small uncontrolled studies examining the acute hemodynamic effects of digoxin in children with heart failure due to large left-to-right shunts showed conflicting results.[16‐18] In a study of 21 infants with heart failure due to ventricular septal defects, Berman *et al*. showed improved contractility in only six patients while 12 improved symptomatically.[16] Kimball *et al*. reported data on digoxin use in 19 infants with ventricular septal defect and found that symptoms and signs were not significantly improved by diuretics or digoxin.[17] There are no data on the efficacy of digoxin in heart failure in children with LV systolic dysfunction or valvar regurgitations and no data on long-term survival in any of these trials.

Despite the lack of data regarding its use in children, digoxin continues to be used by most clinicians in the management of pediatric heart failure due to various causes. The widespread availability, low cost, and continued confidence in the usefulness of the drug based on long years’ of experience are some of the reasons for its continued use. However, it is reasonable to question whether this practice is evidence-based medicine. This opinion poll is an attempt to gather the practice preferences of experts in this field and arrive at a consensus for this debate. The expert opinions on the various issues discussed in the opinion poll are summarized below:

### Is there any role for digoxin in the management of heart failure in children in the current era? If yes, what would be your recommendations indications?

All the experts were unanimous in their opinion that there is a definite role for digoxin in the contemporary management of heart failure in children. All were in agreement that digoxin is clearly indicated in primary myocardial disease with left and/or right ventricular dysfunction. Respondents were divided in their opinion regarding the use of digoxin in other causes of heart failure like left-to-right shunts and valvar regurgitations. While some experts (JMS, BRJK) felt that digoxin is not useful in these situations, most others recommend its use in symptomatic patients.

### When and in what sequence will you initiate digoxin in left-to-right shunts?

Most experts who recommended the use of digoxin in left-to-right shunts preferred to initiate digoxin along with diuretics when the patient became symptomatic. All experts agreed that there is no role for starting digoxin in an asymptomatic infant.

### What is the role of digoxin in the management of valve regurgitations?

Most experts felt that digoxin should be administered only to symptomatic patients with severe valve regurgitation. Dr. Bharat Dalvi added that atrial fibrillation is also an indication, even if the patient is asymptomatic, as the aim would be to control the ventricular rate.

### Should you initiate digoxin therapy in the acute phase in patients with Myocarditis?

Most experts felt it is reasonable to consider adding digoxin in the acute phase of myocarditis. However, it is preferable to start digoxin at a lower dose (½ to ¾ maintenance) in this setting in order to prevent digoxin toxicity. With careful monitoring, the dose maybe increased to maintenance levels.

### What are the indications for rapid digitalization in patients with acute heart failure? What is the recommended protocol for digitalization?

Most experts agreed that acute heart failure due to tachyarrhythmia is a clear indication for rapid digitalization. Some respondents (SSK, JM, SP) felt the need for rapid digitalization in other settings of heart failure as well, provided the patient was not receiving digoxin before. Most experts used the standard oral dosing protocol for rapid digitalization and reserved intravenous administration in selected settings only (poor oral absorption). Some experts (BVD, BRJK) did not recommend rapid digitalization at all and preferred to start maintenance doses from the beginning.

### What are the typical dosing schedules in children? Would you recommend monitoring of drug levels while on maintenance therapy?

Most of the experts felt that there is no need for “Digoxin holidays” in children and the drug can be given seven days a week. Twice-daily administration is recommended for younger patients only (< 10 kg). All experts felt that there is no need for routine monitoring of the drug levels except in specific situations (the use of higher doses, co-administration with other anti-arrhythmic drugs, or in situations where the possibility of toxicity is high, e.g., renal failure).

### What are the situations where the maintenance dose of digoxin is altered?

Renal dysfunction, acute phase of myocarditis, and co-administration with drugs like amiodarone and beta-blockers were situations in which lowering of the maintenance dose was recommended by the experts. Pregnancy (for transplacental therapy of fetal tachyarrhythmia) and management of supraventricular tachyarrhythmia are situations where a higher maintenance dose could be considered, with careful monitoring of the drug levels.

### Would you recommend co-administration of potassium supplements along with digoxin in children?

Most of the experts felt that there is no need for giving potassium supplements routinely to children, unless hypokalemia is documented.

### What are the contraindications for digoxin therapy in children?

Although there were no absolute contraindications for digoxin use in children, all the experts were unanimous in using it judiciously and with caution in those with impaired renal function, hypokalemia, liver disease, ventricular outlet obstruction, and those on amiodarone therapy.

### What are the long-term benefits of digoxin therapy in pediatric heart failure?

Most of the experts felt that digoxin is helpful for control of symptoms and tiding over acute exacerbations of heart failure although there is no effect on long-term survival. Among the individual lesions, most experts were undivided in their opinion about the utility of digoxin in myocardial disease. Some experts (JMS, BRJK) felt that digoxin does not influence outcomes in patients’ left-to-right shunts and valve regurgitations at all.

## CONCLUSIONS

Digoxin continues to play a significant role in the contemporary management of pediatric heart failure, especially in symptomatic patients. Digoxin’s low cost, widespread availability, and the long tradition of use in therapeutic practice make it very appealing for clinicians in developing countries where control of symptoms during the waiting period for surgical intervention is a practically relevant consideration.
